# Deep Q-network-based traffic signal control models

**DOI:** 10.1371/journal.pone.0256405

**Published:** 2021-09-02

**Authors:** Sangmin Park, Eum Han, Sungho Park, Harim Jeong, Ilsoo Yun

**Affiliations:** 1 Department of Transportation System Engineering, Ajou University, Suwon, Republic of Korea; 2 Department of Transportation Engineering Research, Korea Road Traffic Authority, Wonju-si, Republic of Korea; University of Shanghai for Science and Technology, CHINA

## Abstract

Traffic congestion has become common in urban areas worldwide. To solve this problem, the method of searching a solution using artificial intelligence has recently attracted widespread attention because it can solve complex problems such as traffic signal control. This study developed two traffic signal control models using reinforcement learning and a microscopic simulation-based evaluation for an isolated intersection and two coordinated intersections. To develop these models, a deep Q-network (DQN) was used, which is a promising reinforcement learning algorithm. The performance was evaluated by comparing the developed traffic signal control models in this research with the fixed-time signal optimized by Synchro model, which is a traffic signal optimization model. The evaluation showed that the developed traffic signal control model of the isolated intersection was validated, and the coordination of intersections was superior to that of the fixed-time signal control method.

## Introduction

### Background and purpose

Traffic congestion in urban areas is a chronic social problem in modern society, and many urbanized areas suffer from chronic socioeconomic damage due to growing traffic congestion [1]. To solve the traffic congestion problem in urban areas, road capacity can be increased by the construction and expansion of roads, but these alternatives require enormous resources and time. In addition, the number of vehicles on urban roads has increased despite the increase in road capacity through continuous road construction. The reduction in road capacity owing to the operation of traffic signals, which are major traffic control devices in urban areas, may be one of the main causes of traffic congestion in urban areas. If the operation of traffic signals does not reflect the traffic situation, delays may arise due to excessive waiting and may encourage drivers to ignore traffic signal instructions. For the efficient operation of traffic signals, actuated traffic signal controls and various types of adaptive traffic signal control systems, such as SCATS, SCOOT, and COSMOS, have been developed [2, 3]. However, it is difficult for the fixed-time signal control method to respond to fluctuations in vehicle arrival distribution and the unpredictable behavior of drivers at signal intersections [4].

Recently, with the development of artificial intelligence (AI) technology, research to solve complex and difficult problems that exist in reality is increasing. AI technology is prominent in various fields as AI techniques have the advantage of being able to derive the relationship between the factors inherent in complex problems that are difficult to explain. Based on this advantage, there have been increasing attempts to alleviate traffic congestion in downtown areas through the operation of AI-based traffic signals. Therefore, this study aims to develop traffic signal control models using reinforcement learning, to alleviate traffic congestion in signalized urban intersections. Furthermore, the developed traffic signal control model is compared with the existing traffic signal optimization model to evaluate its performance.

### Research scope and process

The spatial scope of this research comprises an isolated intersection and coordinated intersections in an urban area. The content scope is the development of traffic signal control models for isolated and coordinated intersections using AI techniques. To conduct this study, diverse AI techniques were reviewed, and as a result, a reinforcement learning algorithm was selected for traffic signal control.

**Fig 1:**
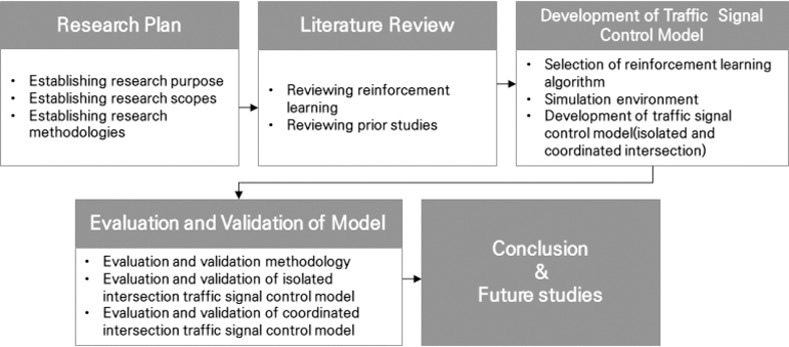
Research process.

In addition, to apply the reinforcement learning algorithm, a simulation environment in which the agents of reinforcement learning can operate was developed. The performance of the developed traffic signal control models was compared with that of the fixed-time signal control method. Conclusions and future research tasks are presented in this paper. Fig 1 illustrates the research process used in this study.

## Literature review

### Deep Q-Network

**Fig 2:**
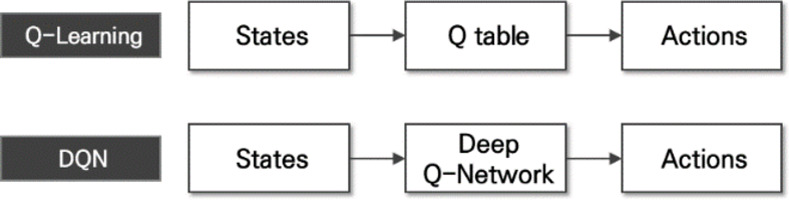
Comparison of Q-learning and DQN.

Reinforcement learning is a machine learning field that solves sequential decision-making problems and is different from supervised and unsupervised learning [5, 6]. Reinforcement learning learns how to behave in a given situation to maximize the reward for [6]. Reinforcement learning consists of three components: actions, reward, and observation. Actions are things that an agent can do in the environment [7]. The reward reflects the success of an agent’s recent action, and it is the first communication channel between the agent and the environment. Observation represents a state of the environment, including a piece of information that the environment provides to the agent [7]. In reinforcement field, there are a variety of algorithms. Especially, a deep Q-network (DQN) is one of the predominant reinforcement learning algorithms designed to overcome the limitations of existing Q-learning algorithms [8]. Q-learning has a Q function that estimates the Q value for each state–action pair and determines whether to perform a specific action in a specific state based on this. The Q function is called an action-value function and can directly estimate the optimal action-value function [6]. In the case of existing Q-learning, the Q value is calculated using the Q function and is stored in the form of a table. However, when the action and state spaces are expressed in the form of a Q table, there is a problem that the cost of calculating the table increases. In contrast, DQN uses a deep neural network (DNN) instead of the Q table to estimate the Q function and compute the Q value. A DQN is suitable for solving problems with large state and action spaces that cannot be estimated with a function or artificial neural network. DQN solves the problem of poor learning in Q-learning based on a function and an artificial neural network (ANN) by using experience replay and target network techniques. Experience replay is a method in which an agent interacts with the environment to obtain an experience and does not immediately use this experience for learning, but instead stores a sample of the experience in memory and randomly extracts the sample in memory for learning. As reinforcement learning’s learning data are generally generated by moving the state space, there is a high correlation between the learning data. However, the use of the experience replay technique has the advantage of removing the high correlation of the training data. This suppresses over-fitting and enables stable learning. The second technique uses the target network to eliminate learning instability when using one network. To calculate the target Q value, the neural network was separated to induce smooth learning. Fig 2 shows the differences between Q-learning and DQN.

### Prior traffic signal control-related studies using reinforcement learning

Li et al. developed a real-time traffic signal control algorithm by integrating Q-learning and deep stacked auto-encoder (SAE) neural networks. The state and reward are defined based on the queue length for each lane. From the simulation analysis, it was found that the proposed algorithm performed better than conventional reinforcement learning. However, the proposed algorithm is limited to the actual situation because it consists only of two-way visions of both through lanes [9].

Van Der Pol studied traffic signal coordination using a DQN. The testbed consisted of a right-angle intersection with a turn prohibition in all directions. For the simulation analysis, Simulation of Urban MObility (SUMO), an open-source-based microscopic traffic simulation software, was used. The state used the location of the vehicle extracted from the image data, and the action consisted of selecting one of the various signal combinations. The reward was a weighted average of several indicators, such as vehicle delay, waiting time, number of stops, and signal changes [10].

Genders and Razavi conducted a traffic signal optimization study using a DQN based on a deep convolutional neural network (CNN). The testbed consisted of a right-angle intersection with four lanes (one left-turn lane, one right-turn lane, and two through-lanes) in each approach. SUMO was used for the simulation, and the state was used to extract the vehicle position from the image and match the matrix. The reward used was the change in the cumulative value of vehicle delay [11].

Jin and Ma performed traffic signal optimization using a reinforcement learning technique that considered each signal group as an agent. The state-action-reward-state-action (SARSA) algorithm was used as a reinforcement learning algorithm. The time gap and occupancy, which can be collected directly from the loop detector, were used as the states. The reward was learned using the average delay. The action was trained using an algorithm that extended every 4 s. The results showed that the proposed algorithm worked better than the traffic signal optimization method based on a genetic algorithm [12].

Gao et al. developed a signal control algorithm by applying a CNN to reinforcement learning. The state data utilized, consisted of the location and speed of vehicles, and the traffic signal indication extracted from the intersection image data. The testbed used a two-phase signal intersection consisting of only two through-lanes. There are two actions: turning on green lights for west–east traffic and for north–south traffic. The reward was defined as the change in the number of vehicles that stopped during the green signal time. The average vehicle waiting time at the intersection converged as learning progressed and the average vehicle lag was reduced, thus showing an excellent performance compared to other signal control methods such as periodic expression [13].

Wang et al. developed an urban traffic signal optimization control strategy using a Q-learning algorithm. The proposed algorithm shows better applicability in complex traffic environments, such as high flow and multiple intersections, but it is not applicable to all traffic conditions [14].

Zheng et al. presented an algorithm for selecting the traffic signal phase using a DQN. The simulation environment was built using SUMO. The state used was the number of vehicles for each movement in a phase and the current signal phase. The action was to choose the phase for the next time interval. The average queue length for each traffic movement was used as a reward. The performance of the proposed algorithm was compared with that of other reinforcement learning methods using the travel time. From the comparison, it was found that the proposed algorithm showed better overall performance than the other methods [15].

Zhao et al. proposed three optimization methods for a junction-tree algorithm (JTA) based reinforcement learning algorithm that can be used for network-wide signal coordination. The first is the existing algorithm. The second is the optimization of the basic parameters and information transmission mode. The third is the optimization of the information transmission rule and the return. It can be concluded that better operational results can be achieved in network-wide signal coordination by applying the proposed optimizations to existing JTA-based reinforcement learning algorithms. However, the reported results are based on a toy network [16].

### Discrimination of research

In previous research, many traffic signal control studies using reinforcement learning were based on the action to maintain the current phase and the action to change to the next phase as a traffic signal control method. In other words, the agent decides whether to keep the current phase or change to the next phase in every predetermined time interval. However, this study proposes two traffic signal control methods. A traffic signal control method selects the signal timing plan (i.e., green splits for individual phases) for the next cycle in advance (i.e., at the end of the current cycle) in response to the intersection status from the signal timing plan lists. Another traffic signal control method is to select a suitable offset for a traffic situation from the offset list. These methods have the advantage of presenting a reliable result because the action selection is made within an acceptable range, which may be defined in the predetermined signal timing and offset lists.

## Development of a traffic signal control model based on a DQN

### Selection and implementation of reinforcement-learning algorithms

In this study, traffic signal control models were developed using reinforcement learning, a machine learning method. Reinforcement learning is an algorithm that solves the problem of sequential decision making to achieve the goal [6]. Reinforcement learning involves various experiences and explorations and has the advantage of not needing to build learning data as a way to learn based on experience. In addition, reinforcement learning is a suitable method for developing a traffic signal control model because it has the advantage of flexibly responding to the situation in the field based on an optimal policy. However, there are various learning algorithms in reinforcement learning, and new algorithms are still being developed. Therefore, it is essential to select an appropriate reinforcement-learning algorithm for this study.

The DQN was selected as a reinforcement learning algorithm because it is widely used in other fields and is considered suitable for developing traffic signal control models. To implement the DQN, Python ver. 3.7, and Pytorch, which is a deep learning library, were used. The DQN implementation was performed using the experience replay memory, deep neural network, learning module, and action selection. The experience replay memory used in this study is one of the functions of a DQN that can lower the correlation of learning data as a space to store enough data for learning. In addition, the experience replay memory size is 50 000 and is implemented to learn when more than 1000 data are stored. In addition, if more than 50 000 data were stored in the experience replay memory, the previous data were deleted and new data were added. In addition, the number of samples used for learning was set to 32, so that the samples were randomly extracted and learned from the data of the experience replay memory.

### Development of simulation environment framework

To perform a DQN, an environment in which the agent can behave and learn itself is essential. To select relevant traffic model, this research reviewed traffic model. There are two groups of traffic flow model which is microscopic models and macroscopic models [17]. The microscopic traffic flow model for an intersection is an important method to explore the mechanism of the influencing factors on the microscopic traffic operations [17].

To develop DQN-based traffic signal control models, a microscopic traffic simulation model was selected as the environment. The microscopic traffic simulation can calculate the position, speed, and acceleration of individual vehicles every second or millisecond. In microscopic traffic simulation, the location of individual vehicles is very precise and realistic because it is calculated by car-following and lane change models that describe the driver’s driving behavior under diverse traffic conditions.

Among the microscopic traffic simulation models, this study selected Vissim, which is capable of controlling traffic signals and obtaining vehicle data through a COM interface. Using Python ver. 3.7, and Vissim COM interface, Vissim-based simulation environment framework was developed, in which DQN algorithms and simulations can interact with each other.

### Development of an isolated intersection traffic signal control model based on DQN

#### Control methodology

To develop an isolated intersection traffic signal control model, it is necessary to determine an appropriate traffic signal control method for reinforcement learning. This study selected a method that provides a signal timing plan corresponding to the traffic conditions for the next cycle in advance. The state is the maximum queue length (i.e., the maximum number of waiting vehicles) for each approach. Here, the maximum queue length is defined as the longest queue length in the cycle. At the end of one cycle, before starting the next cycle, the traffic signal control model selects the optimal signal timing plan based on the state and executes it. Thus, an appropriate signal timing plan is used for each signal cycle, and it is a method of optimizing the isolated intersection traffic signal for each cycle.

**Fig 3:**
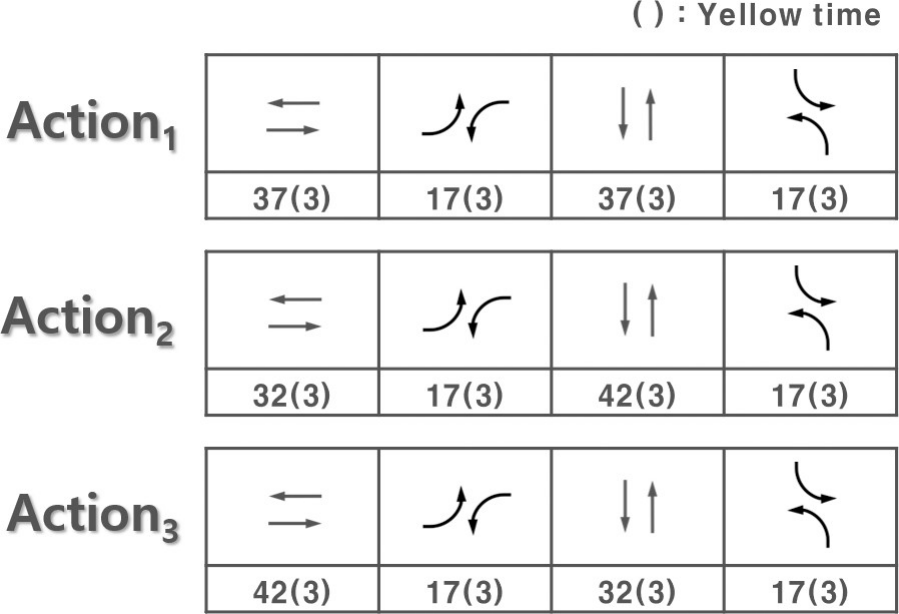
Three predetermined signal timing plan list as actions.

To develop an isolated intersection traffic signal control model using DQN, it is necessary to define the components required for learning. The main components necessary for learning are the state, action, and reward, and are defined according to the isolated intersection traffic signal control model. First, as described above, the maximum queue length per cycle, which can be estimated using a loop detector, was used. To describe the state, a combination of various variables can be considered, but only the maximum queue length is used as the state. The maximum queue length per cycle means the longest queue length per cycle on the approach. Although Avdulhai et al. used the queue length as a state to control phase time, this research utilized maximum queue length per cycle to express the traffic demand as the state on the approaches per cycle because it is needed to control predetermined signal timing plan [18]. Also, the reason for this is the increase in time and resources required for learning because the learning space grows with the addition of variables. Moreover, as an action, the agent selects the signal timing plan of the next cycle at the end of the current cycle. In other words, the agent selects and executes a suitable signal timing plan from the predetermined signal timing plan list for the situation. In this research, three predetermined signal timing plan list to reduce computation time as shown in Fig 3.

**Fig 4:**
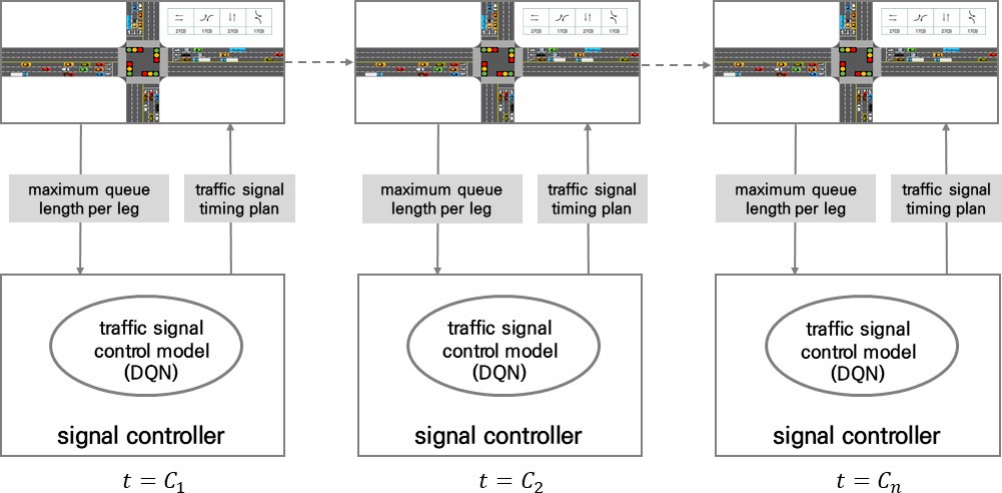
Concept of traffic control method for isolated intersection.

The average stop delay was selected as the reward for the isolated intersection traffic signal control model after literature reviews. Because El-Tantawy et al. used number of stops and delay as a reward, to combine two features into one scalar, average stop delay was used as the reward in this research [19]. That is, the isolated intersection traffic signal control model learns by selecting actions that minimize the average stop delay per cycle. Fig 4 shows the concept of an isolated intersection traffic signal control method.

#### Model development and learning

**Fig 5:**
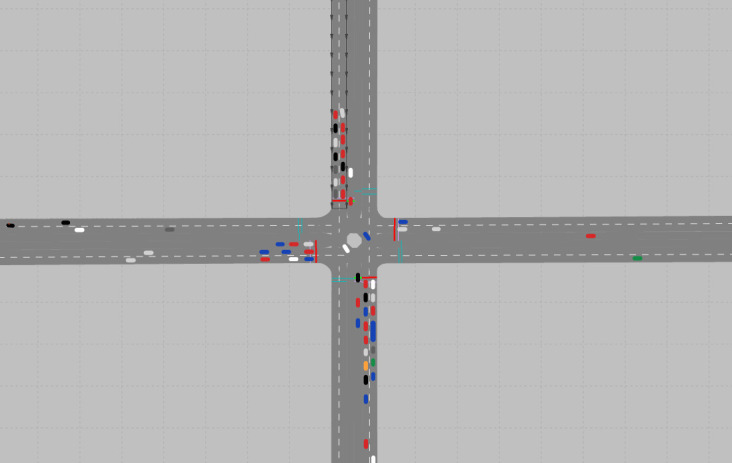
Simulation network of isolated intersection.

The isolated intersection traffic signal control model was developed using a simulation environment, signal control method, and reinforcement learning components for the DQN. In this study, a testbed was constructed using the Vissim software. The testbed is a four-leg intersection with one left-turn lane, one through lane, and one through and right turn lane. The traffic volume composition in each approach consisted of 800 vehicles/hour for through movement, 200 vehicles/hour for left turn, and 100 vehicles/hour for right turn. To make the simulation environment more similar, parameters such as the desired speed distribution and conflict area were considered. In the test bed, the traffic signal control model was trained using the DQN. After learning the 1400 episodes of the model, it was found that the average delay per cycle was constant at approximately 17 s, and the learning was completed. Fig 5 shows the constructed isolated intersection testbed in Vissim.

### Development of coordinated intersection traffic signal control model based on DQN

#### Traffic signal control methodology

To develop a coordinated intersection traffic signal control model, this study selected a methodology that provided an offset corresponding to the traffic conditions for each cycle. To measure the state, the maximum queue length per cycle and travel speed in the coordinated direction were used. This is because the travel speed in the coordinated direction is an important factor in the coordination. The coordinated intersection traffic signal control model is a method for optimizing the offset for each situation based on the traffic speed and queue length.

**Fig 6:**
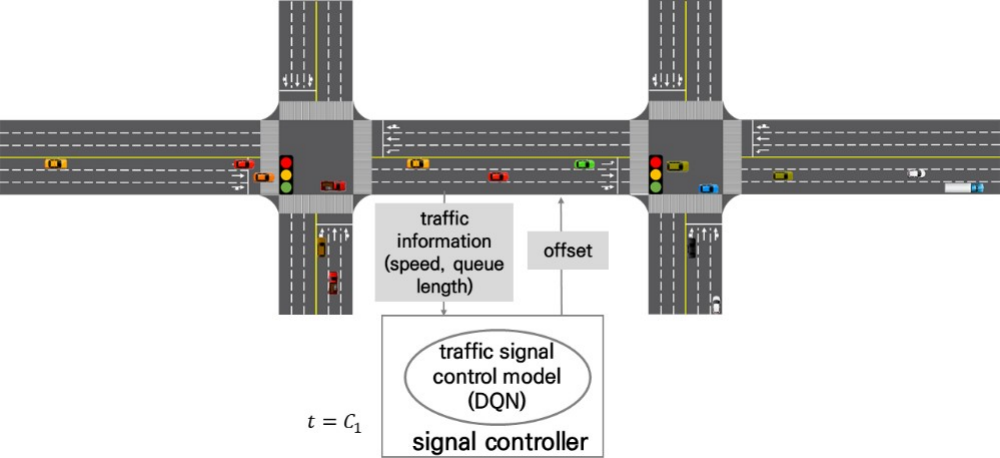
Traffic control method of coordinated intersections.

To develop this control model, important components of reinforcement learning, such as state, action, and reward, were defined. First, to select the state, we examined traffic variables that can be measured using a loop detector from prior studies. In this study, the state was measured using the maximum queue length and travel speed, which can be measured using a loop detector. The offset was chosen as the action selected by the agent in the environment. In traditional signal operation, the offset is set using a signal optimization program, such as Synchro, using the traffic volume and speed data surveyed in the field. However, in actual coordinated intersections, the travel speed may change because of traffic volume changes and other driver behaviors. Such fluctuations can degrade the efficiency of the coordination system operation. Therefore, considering the characteristics of coordinated intersections, it was decided to select an offset as an action and change the offset for each cycle. However, changing the offset can cause a transition problem in coordinated signal control. Therefore, the offset was changed to 1 s. In addition, the offset change was conducted by adjusting the current cycle length through subtraction/addition of the green split of the minor through phase to prevent a transition problem. The offset adjustment started at a predetermined offset value which is 32 s. As with the existing isolated intersection traffic signal control model, the average stop delay is defined as the reward so that the action can be performed in the direction that minimizes it. The coordinated intersection control method is shown in Fig 6.

#### Model development and learning

**Fig 7:**
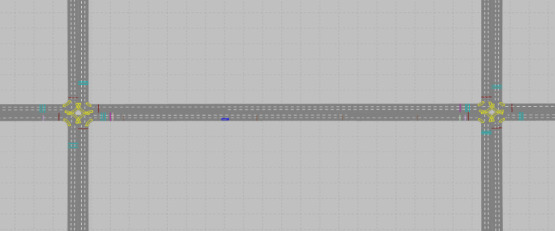
Simulation network of coordinated intersections.

A two-way coordinated intersection network was constructed as a testbed to develop the coordinated intersection traffic signal control model using the DQN. Each intersection in the testbed is a 4-leg coordinated signalized intersection, consisting of one left-turn lane, one through lane, and one through and a right-turn lane. The distance between the two intersections is 400 m, and the speed limit is set to 50 km/h, which is a common speed limit in urban areas in Korea. Fig 7 shows the coordinated intersection-experiment network. In Fig 7, the intersection on the left is intersection 1, and the other is intersection 2.

The east-bound traffic at intersection 1 in Fig 7 consists of 100 vehicles/hour for left-turn, 800 vehicles/hour for through movement, 100 vehicles/hour for right-turn, and 200 vehicles/hour for left-turn, 700 vehicles/hour for through movement, and 100 vehicles/hour for right-turn. The east-bound traffic at intersection 2 consisted of 200 vehicles/hour for left-turn, 700 vehicles/hour for through movement, and 100 vehicles/hour for right-turn, and the west-bound traffic was 200 vehicles/hour for left-turn, 800 vehicles/hour for through movement, and 100 vehicles/hour for right-turn. Intersections 1 and 2 have the same north- and south-bound traffic volumes. The signal phases of intersections 1 and 2 consisted of major road left-turn, major road through movement, minor road left-turn, and minor road through movement. Green splits were set to 27 s for green and 3 s for yellow. After the 980^th^ episode, the learning was completed by repeating the average stop delay per cycle with constant results at a level of approximately 31 s.

## Evaluation and validation of the developed traffic signal control model

### Traffic signal control model evaluation and validation method

#### Selection of MOE

To evaluate the performance of the DQN-based traffic signal control model developed in this study, the measures of effectiveness (MOEs) were selected through a review of prior studies.

From the review, the average stop delay, average delay, average travel speed, and average number of stops were selected as MOEs. The average stop delay is the sum of the times when individual vehicles stop on the network divided by the number of all vehicles in the network. The average delay is the average value of vehicle delays in the network during the simulation experiment. The average travel speed is the value obtained by dividing the total distance traveled by individual vehicles by the total travel time. Finally, the average number of stops refers to the total number of times that individual vehicles stopped while moving divided by the number of all vehicles in the network.

#### Evaluation and validation methodology

The fixed-time signal control method was used as the comparison group to evaluate the performance of the developed DQN-based traffic signal control model. For the isolated intersection traffic signal control model, the fixed-time signal plan optimized using Synchro, which is a signal timing optimization S/W, was used as the comparison group, and for the coordinated intersection traffic signal control model, the offset value optimized using Synchro was used as the comparison group.

In addition, to verify the performance of the proposed model in various situations, a traffic increase and decrease scenario was constructed for the isolated intersection, and an illegal parking situation scenario was used for the coordinated intersections. Applying the defined scenarios, the MOEs of the two models developed in this study and the fixed-time signal plan and offset value optimized using Synchro were compared. For the average stop delay, the Wilcoxon signed-rank test was used to test whether there was a statistically significant difference.

### Evaluation and validation of isolated intersection traffic signal control model

#### Optimization of signal timing plan using Synchro

A comparison group is required to evaluate and validate the developed isolated intersection traffic signal control model. In this study, the optimal signal timing plan was calculated using Synchro 6.0, which is the S/W used to optimize and evaluate the signal timing plan of signalized intersections. As a result, the optimal cycle length was 120 s, and the optimal signal timing plan is presented in Table 1.

**Table 1:** Optimal traffic signal plan calculated by Synchro model.

Phase sequence	Phase 1	Phase 2	Phase 3	Phase 4
**Direction**	North- and southbound lefts	North- and southbound straight through	East- and westbound left	East- and westbound straight through
**Green time**	17 s	37 s	17 s	37 s
**(Yellow time)**	(3 s)	(3 s)	(3 s)	(3 s)

#### Scenarios for evaluation and validation

For evaluation and validation, a 15% traffic volume increase scenario (i.e., scenario 1–2) and a 15% traffic volume reduction scenario (i.e., scenario 1–3) were used. For reference, scenario 1–1, which corresponds to the existing traffic volume, consists of 1100 vehicles/hour in each direction, 800 vehicles/hour for through movement, 200 vehicles/hour for left-turn, and 100 vehicles/hour for right-turn.

#### Results of evaluation and validation

The model was evaluated by comparing the selected MOEs, including the average stop delay in seconds per vehicle, average delay in seconds per vehicle, average speed in kilometers per hour, and average number of stops per vehicle. In the case of the microscopic traffic simulation, as the MOE values calculated according to random numbers are measured differently, this study attempted to improve the accuracy of the analysis through five multi-runs for each scenario.

As presented in Table 2, in scenario 1–1, the performances of the developed isolated intersection traffic signal control model in terms of average delay, average travel speed, and average number of stops did not decrease compared to those of the fixed-time signal plan optimized by Synchro. In addition, the average stop delay was 17.45 s, which was 1.02 s lower than when the fixed-time signal plan optimized using Synchro was used. From the Wilcoxon signed-rank test, the p-value was 0.080 at a significance level of 95%, which was lower than 0.05. The null hypothesis was not rejected. Therefore, it was found that the performance of the isolated intersection traffic signal control model developed in this study guarantee the performance of the optimized signal timing plan using Synchro.

**Table 2:** Evaluation and validation results of scenario 1–1 in isolated intersection.

Model	Average stops delay per cycle	Average delay per cycle	Average speed per cycle	Average number of stops per cycle
**DQN**	17.45	22.01	32.52	0.46
	(0.13)	(0.00)	(0.07)	(0.08)
**Optimal traffic signal timing**	18.47	23.38	31.37	0.49
	(0.38)	(0.01)	(0.26)	(0.28)
**Comparison**	−1.02	−1.37	+1.15	−0.03
**Z-value**	−1.753			
**p-value**	0.080			

(): Standard deviation

As presented in Table 3, where the traffic volume increased by 15%, the average stop delay was 23.08 s, which was 0.19 s lower than the optimal signal timing plan in scenario 1–2. From the Wilcoxon signed-rank test, the null hypothesis was not rejected because the p-value was 0.500 at a significance level of 95%. Therefore, it was found that the performance of the isolated intersection traffic signal control model developed in scenario 1–2 does not deteriorate compared to the performance of the optimized fixed-time signal plan using Synchro. In addition, the performances of the developed isolated intersection traffic signal control model in terms of average delay, average travel speed, and average number of stops could be guaranteed for those of the fixed-time signal plan using Synchro optimization model.

**Table 3:** Evaluation and validation results of scenario 1–2 in isolated intersection.

Model	Average stops delay per cycle	Average delay per cycle	Average speed per cycle	Average number of stops per cycle
**DQN**	23.08	29.01	27.59	0.58
	(0.50)	(0.48)	(0.40)	(0.01)
**Optimal traffic signal timing**	23.27	29.24	27.68	0.61
	(1.09)	(1.38)	(0.72)	(0.04)
**Comparison**	−0.19	−0.23	−0.09	−0.03
**Z-value**	−0.674			
**p-value**	0.500			

(): Standard deviation

In scenario 1–3, presented in Table 4, where the traffic volume was reduced by 15%, the average stop delay was 16.55 s, which was 0.9 s lower than the optimal signal timing plan. Statistical analysis and Wilcoxon signed-rank tests were performed at a significance level of 95%. From the statistical test, the null hypothesis was rejected because the p-value was 0.043. Therefore, it was shown that the performance of the developed isolated intersection traffic signal control model in scenario 1–3 is excellent compared to the performance of the optimal fixed-time signal plan. In addition, the performances in terms of average delay, average travel speed, and average number of stops were superior to those of the optimal fixed-time signal plan.

**Table 4:** Evaluation and validation results of scenario 1–3 in isolated intersection.

Model	Average stops delay per cycle	Average delay per cycle	Average speed per cycle	Average number of stops per cycle
**DQN**	16.55	21.03	32.71	0.44
	(0.09)	(0.11)	(0.07)	(0.00)
**Optimal traffic signal timing**	17.45	22.01	32.52	0.46
	(0.08)	(0.13)	(0.07)	(0.00)
**Comparison**	−0.9	−0.98	+0.19	−0.02
**Z-value**	−2.023			
**p-value**	0.043			

(): Standard deviation

### Evaluation and validation of coordinated intersection traffic signal control model

#### Optimization of offset using Syncrho

The optimal offset value was calculated using Synchro 6.0. Intersection 1 was designated as a master intersection where the offset is ‘0’ s, and the optimal offset value of intersection 2 was set to 32 s.

#### Scenarios for evaluation and validation

The evaluation and validation scenario consists of the scenario used for learning (i.e., scenario 2–1) and the scenario (i.e., scenario 2–2) in which illegal parking on the major road is added. In an illegal parking situation, a vehicle is repeatedly parked on a major road. Illegal parking was realized using a parking function in Vissim. Illegal parking affects the speed of the major road to be coordinated, and thus, the offset value should be adjusted according to the changed travel speed to reflect the real traffic situation.

#### Results of evaluation and validation

The evaluation and verification were performed using the same MOEs used in the isolated intersection case. To set the comparison group in scenario 2–2, the offset value was optimized using Synchro.

Comparing with scenario 2–1, it was found that the developed model has excellent performance in terms of average delay, average travel speed, and average number of stops. In addition, the average stop delay of the developed model was 31.61 s, which was 2.61 s lower than that of the optimal offset value. The Wilcoxon signed-rank test showed a statistically significant difference at a significance level of 95%, as indicated in Table 5.

**Table 5:** Evaluation and validation results of scenario 2–1 in coordinated intersections.

Model	Average stops delay per cycle	Average delay per cycle	Average speed per cycle	Average number of stops per cycle
**DQN**	31.61	37.80	19.51	0.76
	(1.17)	(1.26)	(0.45)	(0.03)
**Optimal traffic signal timing**	34.22	40.69	18.38	0.82
	(2.34)	(2.60)	(1.00)	(0.05)
**Comparison**	−2.61	−2.89	+1.13	−0.06
**Z-value**	−2.023			
**p-value**	0.043			

(): Standard deviation

Comparing the MOEs in scenario 2–2, presented in Table 6, which is an illegal parking situation, the average stop delay was 34.50 s, which was 2.14 s lower than that of the optimal offset. The Wilcoxon signed-rank test showed a statistically significant difference at a significance level of 95%, indicating that the performance was excellent, as summarized in Table 6. In addition, it was confirmed that the performance was superior to that of the optimum offset in terms of average delay, average travel speed, and average number of stops.

**Table 6:** Evaluation and validation results of scenario 2–2 in coordinated intersections.

Model	Average stops delay per cycle	Average delay per cycle	Average speed per cycle	Average number of stops per cycle
**DQN**	34.50	41.22	18.15	0.78
	(0.78)	(0.90)	(0.35)	(0.03)
**Optimal traffic signal timing**	36.64	43.54	17.24	0.88
	(1.30)	(1.55)	(0.56)	(0.06)
**Comparison**	−2.14	−2.32	+0.91	−0.1
**Z-value**	−2.023			
**p-value**	0.043			

(): Standard deviation

## Conclusions

To solve traffic congestion in urban areas, isolated intersection and coordinated intersection traffic signal control models were constructed using DQN, an emerging reinforcement learning technology that offers a solution to complex and diverse problems. Before constructing the two models, reinforcement learning and related prior studies were reviewed. Based on this, the DQN algorithm, which shows high performance among the reinforcement learning algorithms, was selected and implemented. In addition, because an external environment is required for reinforcement learning, a simulation environment was constructed using Vissim and the COM interface. Subsequently, the traffic signal control method of the isolated and coordinated intersections was determined and learned to find the optimal policy through reinforcement learning to complete the development of the traffic signal control model. Iterative experiments were performed using a microscopic traffic simulation in the scenarios constructed to evaluate and verify the completed models. In the case of the developed isolated intersection traffic signal control model, the performance was guaranteed those of the fixed-time signal control method optimized by Synchro optimization model in the existing and traffic increase and decrease scenarios.

In addition, it was confirmed that the developed coordinated intersection traffic signal control model performs better than the offset using Synchro in both scenarios. This is considered to be the result of the higher effect of reinforcement learning in complex or uncertain situations.

In this study, a traffic-signal control model is developed using reinforcement learning. However, additional research is needed because of the limitations of this study. First, the model was developed and evaluated using a simple microscopic traffic simulation-based testbed. The actual road environment has many diverse and uncertain factors, but the testbed applied is so simple that it reflects real road status. Thus, it may be difficult to apply the developed traffic signal control model to a real site. It is necessary to conduct research that considers more complicated real situations. In addition, isolated intersection and coordinated intersection traffic signal control models were developed and evaluated. In the case of traffic signal control, the cycle, phase, signal time, and offset must all be considered. Because the signal timing plan and offset are closely related, it is ideal to calculate the traffic signal control variables in consideration of both the offset, signal timing plan, and cycle when traffic increases. Therefore, in the future, it will be necessary to develop a traffic signal control model that considers and calculates various traffic signal control variables, such as cycle, phase, signal time per phase, and offset. Finally, the developed models were not compared with other existing traffic signal control methods, including traffic response signals and real-time traffic control systems. In the future, it will be necessary to compare and evaluate the effects of various real-time traffic control systems, such as COSMOS, SCATS, and SCOOT.

## Supporting information

S1 Data (ZIP)
